# The mediating role of sleep quality in the association between urinary incontinence and depressive symptoms: A study with older Korean Americans Living in Subsidized Senior Housing

**DOI:** 10.1371/journal.pone.0354131

**Published:** 2026-07-30

**Authors:** Seo-Yun Choi, Ju Hyun Kim, Soondool Chung, Yuri Jang

**Affiliations:** 1 Edward R. Roybal Institute on Aging, Suzanne Dworak-Peck School of Social Work, University of Southern California, Los Angeles, United States of America; 2 Department of Sociology, Chungnam National University, Daejeon, Republic of Korea; 3 Department of Social Welfare, Ewha Womans University, Seoul, Republic of Korea; Ladoke Akintola University of Technology Teaching Hospital: LAUTECH Teaching Hospital, NIGERIA

## Abstract

Urinary incontinence (UI) is a prevalent health issue among older adults. Depressive symptoms often accompany UI; however, the pathways linking these conditions remain understudied. In this study, we conceptualized sleep quality as a potential mediator in the association between UI and depressive symptoms. The mental health burden of UI is anticipated to be indirectly associated with impaired sleep quality. The mediating role of sleep quality was tested in a sample of 306 older Korean Americans living in low-income senior housing (mean age = 79.4 years, range = 65–102). Regression analyses showed a significant association between UI and depressive symptoms. Mediation analysis using the Hayes PROCESS macro showed that the indirect effect of UI on depressive symptoms through sleep quality was significant (B [SE] = 0.59 [0.21], bias-corrected 95% CI = [0.20, 1.02]), accounting for approximately 24% of the total effect. The findings suggest that compromised sleep quality may serve as a potential pathway linking UI and depressive symptoms. Sleep quality should be addressed in efforts to promote the mental health of older adults experiencing UI.

## Introduction

Defined as the unintentional or involuntary leakage of urine [1,2], urinary incontinence (UI) has been identified as one of the common geriatric conditions [3]. About 30% of community-dwelling older adults worldwide are known to be affected by UI [4], and among US Medicare beneficiaries, more than 11% are diagnosed with UI [5]. Often leading to physical and emotional discomfort, low self-esteem, and social alienation [6–8], UI has negative impacts on mental health. Particularly the link between UI and depressive symptoms is well-established [4,6,9,10]; however, the underlying mechanism remains largely unexplored.

One potential construct that may help explain the association between UI and depressive symptoms is sleep quality. UI exacerbates sleep disruption by placing additional strain on a sleep environment often compromised in later years of life. In particular, nocturia causes frequent awakenings during sleep, ultimately disrupting sleep patterns [11,12]. Studies suggest that UI is associated with an increased risk of sleep disturbances, with some evidence of a dose-response relationship between UI severity and sleep disruption [13]. Studies also report a close connection of UI with poor sleep position and interrupted sleep duration [14].

Sleep is also strongly associated with mental health [15]. Poor sleep quality has shown to have a direct impact on the occurrence and exacerbation of depressive symptoms [16–18]. A longitudinal study over 10 years found that older adults with persistent sleep problems had a 2–3 times higher risk of developing depression compared to those without [19]. Other studies have identified sleep disturbances as a strong predictor of depression relapsing, suggesting the neurobiological link between sleep and emotional regulation [15,20]. Such independent links of sleep quality with UI and depressive symptoms elucidate the potential of sleep quality to serve as an intermediary.

The mediating role of sleep quality in the association between various life stressors and depressive symptoms has been well-documented. For example, studies have reported that stressful life events such as bereavement, cognitive decline, and caregiving can increase depressive symptoms by making individuals more susceptible to sleep disturbances [16 21]. In a study on physical health constraints [22], the indirect effect of chronic diseases on depressive symptoms through sleep disturbance was found to be significant. However, the model was tested using a global sum of chronic diseases, without considering specific type of the conditions. The mediating role of sleep quality in the association between UI and depressive symptoms has rarely been examined.

This study focuses on Korean American older adults residing in subsidized senior housing. Koreans are the fifth-largest Asian subgroup in the United States, and the Los Angeles area is home to the largest Korean American population [23]. Older Korean Americans living in subsidized senior housing have been shown to be particularly vulnerable to depressive symptoms, due to acculturative stress, physical health issues, limited socioeconomic resources, and weakened social networks of family and friends [9,17,24]. Previous studies demonstrated high prevalence rates of UI and sleep problems in older Koreans residing in low-income senior housing and their negative impacts on various aspects of health and well-being [9,17]. However, the dynamics among UI, sleep quality, and depressive symptoms have not been explored. Addressing this gap, we hypothesize that the negative mental health impact of UI will be indirect through impaired sleep quality. The mediating role of sleep quality will inform strategies for enhancing mental health of older adults affected by UI.

## Methods

### Sample

The data for the present study were drawn from the cross-sectional surveys with Korean-American residents in subsidized senior housing in the greater Los Angeles area. After obtaining approval from the University of Southern California Institutional Review Board (UP-23–00115), survey data were collected from April through June 2023. The structured 10-page questionnaire in Korean was designed to be self-administered; however, bilingual and bicultural survey assistants were available for those who needed help. A total of 351 participants were surveyed across six facilities located within a 20-mile radius of the research site. Surveys were conducted in common areas of the facilities (e.g., meeting rooms and cafeterias) to provide a comfortable environment for participants. Surveys were open to Korean Americans 62 years of age or older, residents of participating facilities, and with the ability to understand and sign a consent form. Following the completion of the self-administered survey, trained research personnel assessed participants’ cognitive function using the Mini Mental State Examination (MMSE). Participants with severe cognitive impairment (MMSE ≤ 10) were excluded from the study to ensure data reliability. After deleting participants with missing data on the variables used in the present investigation, the final sample size was 306.

### Measures

***Urinary incontinence (UI)***. Participants were asked to report the frequency of involuntary urine leakage or loss of bladder control over the past 12 months on a 4-point scale (0 = never, 1 = seldom, 2 = sometimes, 3 = often). Due to the skewed distribution of responses, the variable was recoded into a binary indicator of UI experience (0 = no UI experience; 1 = any UI experience) to improve model stability and interpretability.

***Sleep quality.*** Sleep quality was assessed using the question: “How would you rate your sleep?” Responses were recorded on a 5-point scale ranging from 1 (poor) to 5 (excellent). This single-item self-rating has been shown to be a valid and efficient measure of overall sleep quality [25,26].

***Depressive symptoms.*** Depressive symptoms were measured using the Patient Health Questionnaire–9 (PHQ–9) [27]. Participants were asked to report how often they were bothered by problems such as ‘little interest or pleasure in doing things,’ ‘feeling down, depressed, or hopeless,’ and ‘poor appetite or overeating’ over the past 2 weeks. Each item was rated on a 4-point scale, ranging from 0 (not at all) to 3 (nearly every day). Total scores could range from 0 to 27, with higher scores indicating greater levels of depressive symptoms. The scale has been translated into Korean, and the psychometric properties of the translated version have been validated [28]. In this sample, the scale demonstrated high internal consistency (α = .86). Given that the PHQ-9 includes an item assessing sleep problems (i.e., trouble falling or staying asleep, or sleeping too much), this potential overlap was taken into consideration in the analytic plan.

***Covariates.*** Guided by the literature on aging populations and immigrant health [9,17], a set of background variables representing socio-demographic characteristics and health status was selected as covariates. Sociodemographic characteristics selected were age (in years), sex (0 = male, 1 = female), marital status (0 = not married, 1 = married), educational attainment (0 = ≤ high school graduation, 1 = > high school graduation), length of stay in the United States (in years), and chronic medical conditions. Chronic medical conditions are considered as a general indicator of physical health status. The total count for the checklist of 10 chronic diseases and conditions common among older adults (e.g., diabetes, cancer, arthritis, heart disease, high blood pressure) was used as a continuous format.

### Analytic strategy

After reviewing the descriptive statistics, we examined bivariate correlations among the study variables to assess their associations and ensure the absence of collinearity. We then conducted multivariate linear regression analysis to test the direct association of UI with sleep quality on depressive symptoms. In the first model, the effect of UI was tested after controlling for the set of covariates (age, sex, marital status, education level, length of residence in the United States, and chronic medical conditions). Subsequently, sleep quality was added to the model, which was intended to examine not only its direct effect but also the possibility of its role as a mediator (i.e., whether the magnitude of the impact of UI in the first model would be reduced when sleep quality was included). The mediation hypothesis was confirmed by the Hayes’ PROCESS macro, which tested the indirect effect model of UI on depressive symptoms through sleep quality. We used 5000 bootstrap samples and bias-corrected 95% confidence intervals (CI). The CIs excluding zero would indicate the statistical significance of the indirect effect [29]. To account for the potential overlap between the sleep measure and the sleep-related item included in the PHQ-9, sensitivity analyses were conducted using PHQ-9 scores with the sleep item removed. These analyses were performed to determine whether the observed findings were robust to the exclusion of sleep-related content from the depression measure. All analyses were performed using IBM SPSS Statistics 29 (IBM Corp., NY).

## Results

### Descriptive characteristics of the sample

Table 1 summarizes sample characteristics. The mean age of the participants was 79.4 years (*SD* = 6.65), and about 71.6% were female. About 40% of the participants were married and 37.6% were higher than high school graduation. All participants were foreign-born, and the length of residence in the United States averaged 32.5 years (*SD* = 11.1). On average, the sample had 1.76 chronic medical conditions (*SD* = 1.19). More than half of the sample (57.2%) reported experiencing UI during the previous 12 months. The mean scores for sleep quality and depressive symptoms were 2.61 (*SD* = 1.17) and 5.85 (SD = 4.68), respectively.

**Table 1:** Descriptive Characteristics of the Sample (n = 306).

	Value
Urinary incontinence (UI), %	57.2
Sleep Quality, M ± SD	2.61 ± 1.17
Depression symptoms, M ± SD	5.85 ± 4.68
Age, M ± SD	79.4 ± 6.65
Female, %	71.6
Married, %	39.5
>high school graduation, %	37.6
Year in the U.S., M ± SD	32.5 ± 11.1
Chronic medical conditions, M ± SD	1.76 ± 1.19

*Note.* Binary variables were coded as follows: UI experience (0 = no UI experience, 1 = any UI experience), sex (0 = male, 1 = female), marital status (0 = not married, 1 = married), and educational attainment (0 = high school graduation or less, 1 = more than high school education).

### Bivariate correlations of the study variables

As shown in Table 2, experiencing UI was significantly associated with poorer sleep quality and higher levels of depressive symptoms. Sleep quality was also negatively correlated with depressive symptoms. Older age was associated with a greater likelihood of reporting UI and with higher levels of depressive symptoms. Those with higher educational attainment were likely to report better sleep quality and fewer depressive symptoms. Chronic medical conditions emerged as a common correlate of UI experience, sleep quality, and depressive symptoms; those with more numbers of conditions had a greater likelihood of experiencing UI, poorer quality of sleep, and more frequent symptoms of depression. The highest correlation coefficient was found in the association between sleep quality and depressive symptoms (*r* = −.43, *p* < .001), and there was no concern about collinearity.

**Table 2:** Bivariate Correlations among Study Variables.

	1	2	3	4	5	6	7	8	9
1. UI experience	–								
2. Sleep quality	−.22***	–							
3. Depression symptoms	.32***	−.43***	–						
4. Age	.12 *	−.08	.18**	–					
5. Sex (Female)	−.08	−.00	−.02	−.04	–				
6. Marital status (Married)	−.02	.04	−.08	−.09	−.26***	–			
7. Education (>high school graduation)	−.08	.13*	−.14*	−.09	−.27***	.20***	–		
8. Year in the U.S.	−.01	.01	−.10	.01	−.04	−.01	.07	–	
9. Chronic medical conditions	.19**	−.20***	0.25***	.08	.13*	−.13*	−.19***	−0.02	–

*p < .05. **p < .01. ***p < .001.

### Multivariate regression models of depressive symptoms

Table 3 presents the regression models for depressive symptoms. The direct effect of UI was significant (B [SE] = 2.46 [.51], p < .001) even after covariates were controlled. The initial model accounted for 17% of the variance of depressive symptoms. In the subsequent model, the entry of sleep quality added 11% of the variance to the model, resulting in a total explained variance of 28%. Both UI experience (B [SE] = 1.88 (.49), p < .001) and poor quality of sleep (B [SE] = −1.40 [.21], p < .001) were associated with greater levels of depressive symptoms. It was notable that the impact of UI was reduced when sleep quality was included in the model. The overall pattern suggested the potential mediation effect of sleep quality.

**Table 3:** Direct effect models for depressive symptoms.

	B (SE)	
Urinary incontinence	2.46 (.51)***	1.88 (.49)***
Sleep quality	–	−1.40 (.21)***
Age	.07 (.04)	.07 (.04)
Female	−.52 (.59)	−.43 (.55)
Married	−.43 (.53)	−.43 (.49)
>High school graduation	−.73 (.54)	−.43 (.51)
Length of stay in the U.S.	−.04 (.02)	−.04 (.02)
Chronic medical conditions	.68 (.22)**	.48 (.20)*
R²	.17***	.28***
Changes in R²	–	.11***

* *p* < .05. ** *p* < .01. *** *p* < .001.

### The mediation effect of sleep quality

**Fig 1:**
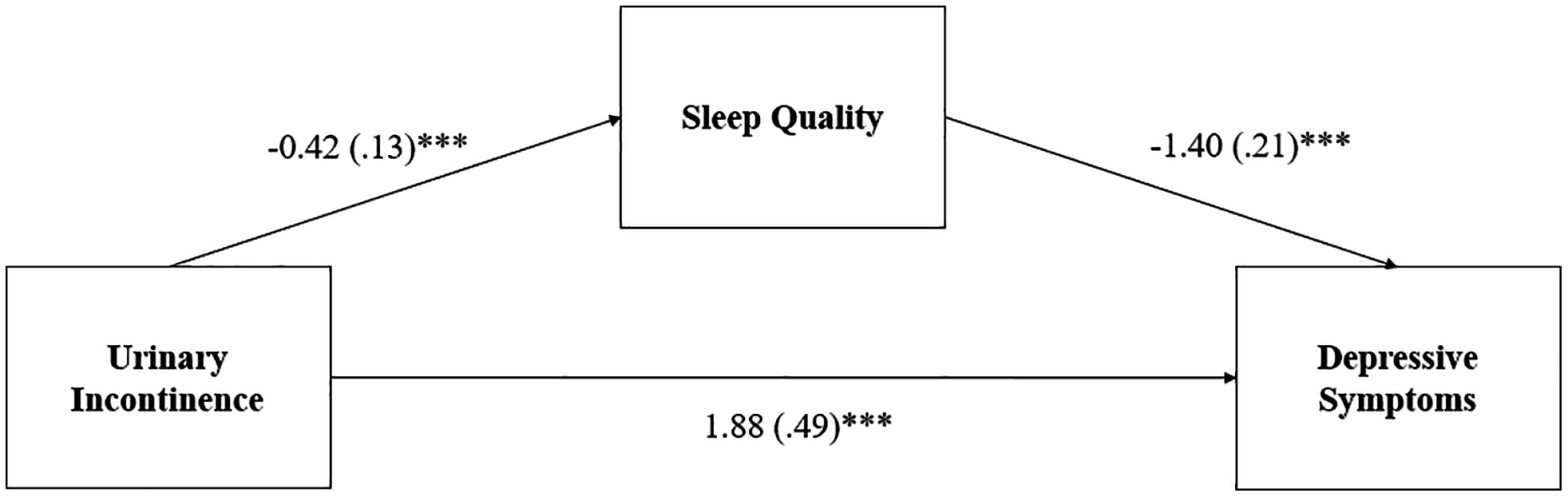
Mediation model of sleep quality. Indirect effect of UI experience on depressive symptoms through sleep quality = 0.59 (0.21), Bias corrected 95% CI for the indirect effect (0.20, 1.02). Age, sex, marital status, education level, length of residence in the United States, and chronic medical conditions were controlled for the analysis. (*** *p* < .001.).

We further examined the mediating role of sleep quality in the association between UI experience and depressive symptoms. As illustrated in Fig 1, the indirect effect of UI experience on depressive symptoms through sleep quality was significant, as indicated by the bias-corrected 95% bootstrap CI, not including zero (B [SE] = 0.59 [0.21], bias-corrected 95% CI = [0.20, 1.02]). In the sensitivity analysis excluding the sleep-related item from the PHQ-9, the indirect effect remained significant (B [SE] = 0.43 [0.17], 95% CI = [0.14, 0.79]), supporting the robustness of the indirect association.

## Discussion

UI is a common geriatric condition that is widely recognized for its negative impact on mental health [3]. Given that the underlying mechanisms linking UI to mental health outcomes remain understudied, the present study conceptualized sleep quality as a potential mediator between UI and depressive symptoms. Our assessment focused on Korean American older adults residing in subsidized senior housing— an immigrant population characterized by socioeconomic disadvantages and health disparities [30]. Our analyses revealed not only the high prevalence of UI, impaired sleep quality, and depressive symptoms within this group, but also an indirect effect of UI experience on depressive symptoms through sleep quality.

In line with previous studies with residents in low-income senior housing [31], our sample demonstrated adverse health profiles. The prevalence of UI was notably high, being experienced by more than half of the sample. The sample’s mean depressive symptom score was also higher than those observed in other sample of older adults [32]. When the PHQ-9 cut-off score was used, more than 18.3% fell into the category of moderate to severe depression, and this rate is considerably higher than the 8.0% reported in the general U.S. older population [33]. Sleep quality was also found to be unfavorable; almost half of the sample reported their sleep as either fair or poor, which is considerably higher than the rate observed in other samples of older adults [24,25]. These findings call attention to low-income senior housing residents’ elevated risks for physical, mental, and sleep health.

Our direct effect models confirmed findings from previous studies on each independent path: the linkages between UI and depressive symptoms [4], between UI and sleep quality [34], and between sleep quality and depressive symptoms [15]. Advancing this knowledge, our indirect effect model provided support for the hypothesis that the association between UI and depressive symptoms would be mediated by sleep quality. Although causal inferences are limited by the cross-sectional design, the findings provide preliminary support for a mediational model in which sleep quality may be associated with the relationship between UI experience and depressive symptoms. Longitudinal research is needed to clarify the temporal ordering of these associations.

The mediating role of sleep quality in the association between physical and mental health has been reported previously [14]. A unique contribution of the present study is its focus on UI as an important factor associated with both poor sleep quality and mental well-being. The identification of the indirect pathway where sleep quality serves as an intervening step not only helps us better understand the association between UI and depressive symptoms but also provides implications for interventions. Given that both UI and depression are the conditions that are subject to cultural and social stigmatization [35,36], sleep may offer safe and acceptable avenues for treatments and services. Such approaches would be particularly relevant in the senior housing context where privacy can be rather easily compromised.

Several limitations should be considered when interpreting findings. As discussed earlier, the foremost concern is the use of a cross-sectional design, which limits our ability to draw causal inferences. Future studies should use a longitudinal design to address the sequential dynamics among UI, sleep quality, and mental health. It should also be noted that both UI and sleep quality were measured using a single-item self-report. Although validated, these measures may not fully capture the complex and multidimensional nature of UI or sleep quality. In addition, UI was assessed based on experiences over the past 12 months, whereas the sleep measure did not specify a particular reference period, which should be considered when interpreting the findings. Future studies should use more comprehensive and time-aligned measures to better capture the dynamic and multidimensional nature of UI and sleep quality, as well as their temporal relationships. It should also be noted that the volunteer nature of sample recruitment may have excluded older individuals with mobility challenges and those who are socially isolated. Although a significant amount of variance in depressive symptoms was accounted for by the proposed model, other contextual factors such as medication use, mobility limitations, pain, and social isolation should be considered in future studies. Despite these limitations, sleep quality emerged as an important factor in the association between UI and mental health, suggesting that interventions targeting sleep hygiene and nighttime UI management may be relevant for individuals affected by UI.

## Supporting information

S1 File Dataset deidentified. (XLSX)

S2 File Inclusivity questionnaire. (DOCX)
